# Complete Chloroplast Genome Sequence of an Engelmann Spruce (*Picea engelmannii*, Genotype Se404-851) from Western Canada

**DOI:** 10.1128/MRA.00382-19

**Published:** 2019-06-13

**Authors:** Diana Lin, Lauren Coombe, Shaun D. Jackman, Kristina K. Gagalova, René L. Warren, S. Austin Hammond, Helen McDonald, Heather Kirk, Pawan Pandoh, Yongjun Zhao, Richard A. Moore, Andrew J. Mungall, Carol Ritland, Trevor Doerksen, Barry Jaquish, Jean Bousquet, Steven J. M. Jones, Joerg Bohlmann, Inanc Birol

**Affiliations:** a Canada’s Michael Smith Genome Sciences Centre, BC Cancer, Vancouver, BC, Canada; b Department of Forest and Conservation Sciences, University of British Columbia, Vancouver, BC, Canada; c British Columbia Ministry of Forests, Lands and Natural Resource Operations, Tree Improvement Branch, Kalamalka Forestry Centre, Vernon, BC, Canada; d Forest Research Centre and Institute for Systems and Integrative Biology, Université Laval, Québec City, QC, Canada; e Michael Smith Laboratories, University of British Columbia, Vancouver, BC, Canada; University of California, Riverside

## Abstract

Engelmann spruce (Picea engelmannii) is a conifer found primarily on the west coast of North America. Here, we present the complete chloroplast genome sequence of *Picea engelmannii* genotype Se404-851. This chloroplast sequence will benefit future conifer genomic research and contribute resources to further species conservation efforts.

## ANNOUNCEMENT

We sequenced, assembled, and annotated the complete chloroplast genome of Engelmann spruce (Picea engelmannii, genotype Se404-851). The Engelmann spruce dominates much of the large spruce forests of interior British Columbia, where it has been reported to hybridize with Picea glauca and Picea sitchensis (1), and its range extends southward to New Mexico. The tree has three different genomes, a nuclear genome, a mitochondrial genome, and a plastid genome (i.e., chloroplast). In general, chloroplast genomes are derived from the ancestral genomes of the microbial endosymbiont from which these organelles originated (2).

A tissue sample was collected from a 13-year-old Engelmann spruce grown at the Kalamalka Forestry Centre in British Columbia (50°14′38.4ʺN, 119°16′40.8ʺW; elevation, 450 m) and planted from a seed from Don Fernando Mountain, New Mexico (36°17′60ʺN, 105°24′0ʺW; elevation, 2,987 m). Genomic DNA was extracted from 60 g tissue by Bio S&T using an organelle exclusion method yielding 300 μg of high-quality purified nuclear DNA, as previously described (3). The sample was sequenced at Canada’s Michael Smith Genome Sciences Centre.

To sequence the sample, a 900-bp whole-genome library was constructed following a previously described protocol (4, 5) with minor modifications. Briefly, 5 μg of genomic DNA was subjected to shearing by sonication (Covaris LE220) using a duty factor of 5 and peak incident power of 450 for 70 seconds. The sonicated DNA products were fractionated in a 6% PAGE gel to recover fragments greater than 700 bp for library preparation. These PCR-free libraries were sequenced with paired-end 150-base reads on an Illumina HiSeq X platform using V4 chemistry according to the manufacturer’s recommendations. With this protocol, four libraries were generated, sequencing approximately 200 million reads from each of them.

To assemble the chloroplast genome, we subsampled the whole-genome shotgun sequencing reads of one lane of one library (i.e., 41,748,620 read pairs) to subsets of 0.75, 1.5, 3, 6, 12, 25, and 41 million read pairs and then assembled each subset with ABySS v2.1.1 (6) (*k*-mer size [*k*], 128; *k*-mer count [*kc*], 3). The ABySS assembly of the 3-million read-pair subset resulted in a single 123,601-bp contig that aligned to the reference chloroplast sequence (*Picea glauca* admix genotype PG29, NCBI accession number NC_028594 [7]), with zero misassemblies and internal gaps, based on QUAST v5.0.0 (8) analysis.

Using BLAST v2.7.1 (9), we aligned our assembly to the reference chloroplast sequence (PG29), modifying start and stop positions for consistency with previously published conifer chloroplast genomes. To ensure that there were no missing sequences at the ends of our assembly, we introduced a gap at the end, circularized the sequence, and ran Sealer v2.1.1 (10), closing the “end” gap and removing overlapping sequences as previously described (11). Finally, the resulting assembly was polished using Pilon v1.22 (12) using the 3-million subset of read pairs aligned with the Burrows-Wheeler Aligner (BWA) v0.1.7 (13).

**FIG 1:**
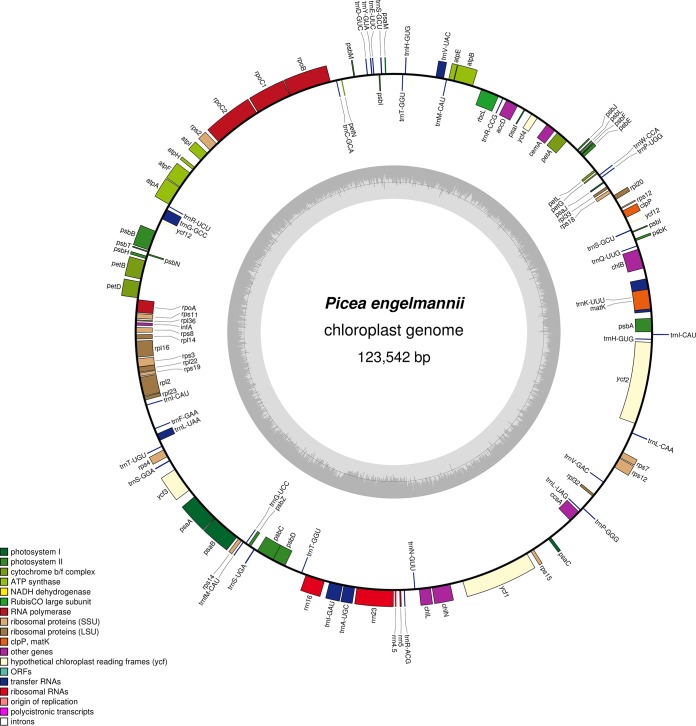
Complete chloroplast genome of Picea engelmannii genotype Se404-851. The *Picea engelmannii* chloroplast genome was annotated using GeSeq v1.65 (14) and plotted using OrganellarGenomeDRAW v1.2 (15). The inner gray circle illustrates the GC content of the genome.

The complete *P. engelmannii* genotype Se404-851 chloroplast genome is 123,542 bp long with a 38.74% GC content. Using GeSeq v1.65 (14) and using other *Picea* chloroplast genomes as references (7, 11), we annotated 114 genes comprising 74 protein-coding genes, 36 tRNA-coding genes, and 4 rRNA-coding genes. We note that four genes (*rps12*, *petB*, *petD*, and *rpl16*) in this list were manually annotated. We used OrganellarGenomeDRAW v1.2 (15) to generate the map in Fig. 1.

The introduction of this new chloroplast genome will benefit conifer genomic research and inform future evolutionary studies.

### Data availability.

The complete chloroplast genome sequence of *Picea engelmannii* genotype Se404-851 is available under GenBank accession number MK241981, and the raw reads are available under SRA numbers SRX5070635 and SRR8252852. The annotations used as references were from Picea abies (GenBank accession number NC_021456), Picea asperata (GenBank accession number NC_032367), Picea glauca genotype PG29 (GenBank accession number NC_028594), Picea morrisonicola (GenBank accession number NC_016069), and Picea sitchensis (GenBank accession numbers NC_011152 and KU215903).
